# Oligodendroglioma of the ciliary body: a unique case report and the review of literature

**DOI:** 10.1186/1471-2407-10-579

**Published:** 2010-10-23

**Authors:** Qing Guo, Jie Hao, Shou bin Sun, Shou ping Xu, Qian Yang, Qi liang Guo, Guo dong Cui

**Affiliations:** 1 Department of life and engineering, Harbin Institute of Technology, Harbin, China; 2 Department of Ophthalmology, the First Clinical Medical School of Harbin Medical University, Harbin, China; 3 Optometry Clinic, the Forth Clinical Medical School of Harbin Medical University, Harbin, China; 4 Department of General Surgery, the First Clinical Medical School of Harbin Medical University, Harbin, China; 5 Department of Ophthalmology, Fourth Hospital of Harbin City, Heilongjiang Province, Harbin, China; 6 Department of Ophthalmology, the Fifth Clinical Medical School of Harbin Medical University, Daqing, China

## Abstract

**Background:**

To date, there is no report in the international literature of an oligodendroglioma of the ciliary body, nor is there an analysis of the possible origins of this lesion.

**Case presentation:**

Here we report on a 52-year-old man admitted to our hospital with a ciliary body tumor revealed by clinical examination and ultrasound, computed tomography and magnetic resonance imaging studies. Following enucleation, pathological and immunohistochemical analyses were performed. Postoperative histopathological staining results included OLIGO-2(+) and GFAP(-), leading to a pathological diagnosis of oligodendroglioma of the ciliary body in the right eye (WHO grade II).

**Conclusions:**

Since malignant gliomas derive from transformed neural stem cells, the presence of oligodendroglioma in the ciliary body supports the hypothesis that gliomas can occur wherever neural stem cells exist. Tumors of the ciliary body derived from oligodendrocytes are difficult to diagnose; pathological analyses are essential.

## Background

The list of most commonly occurring primary brain tumors includes gliomas, and the vast majority of these are astrocytomas or oligodendrogliomas. The latter usually originate from oligodendrocytes in the brain parenchyma, especially in adult patients. To our knowledge,the oligodendroglioma of the retina [1,2] and the ciliary body astrocytoma [3-6] have been reported in the literature, but the oligodendroglioma of the ciliary body has not been reported, nor is there an analysis of the possible origins of this lesion. Here we describe the first case of this tumor type occurring in the right ciliary body of a patient who presented with severely compromised vision.

## Case presentation

**Figure 1:**
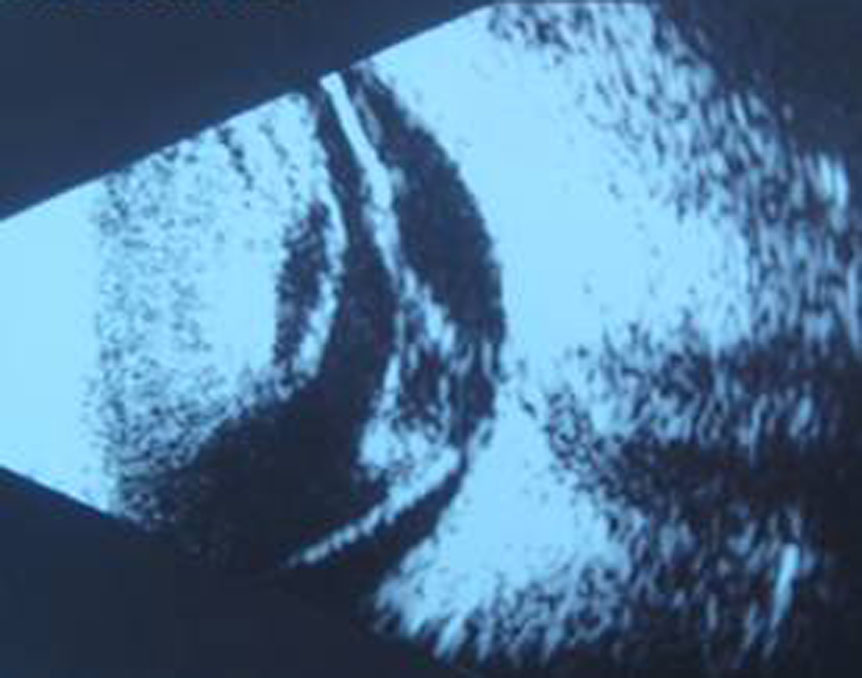
**A mass-like echo, medium intensity, roughly distributed within the echo in the right anterior vitreous cavity**. The posterior vitreous revealed a "v"-shaped echo.

**Figure 2:**
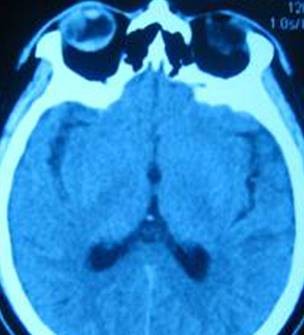
**Computed tomography scan reveals a semi-circular, non-homogeneous, high-density area in the right eye extending from the vitreous cavity to the concave surface of the outer ring, and a crescent-shaped shadow with a clear border in the posterior vitreous**.

**Figure 3:**
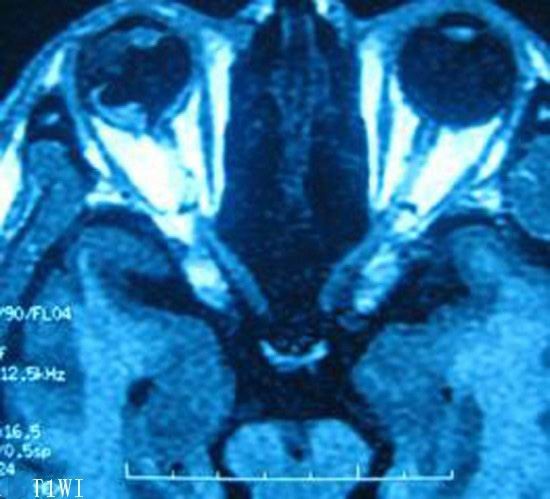
**Magnetic resonance imaging revealed a block with irregular and unclear edges and a long T1 signal area in the lateral ciliary body**. The signal was equal to that from the brain cortex.

**Figure 4:**
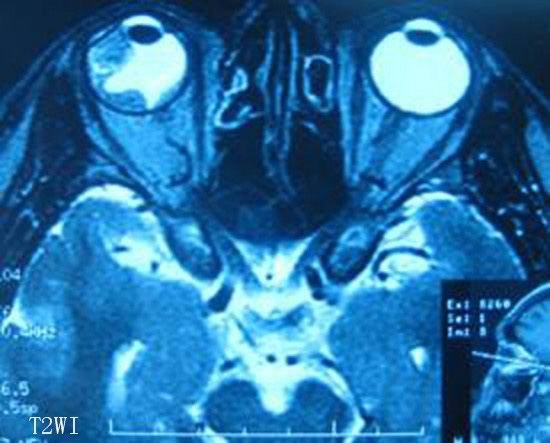
**Magnetic resonance imaging revealed a block with irregular and unclear edges and a short T2 signal area in the lateral ciliary body**. The signal was equal to that from the brain cortex.

**Figure 5:**
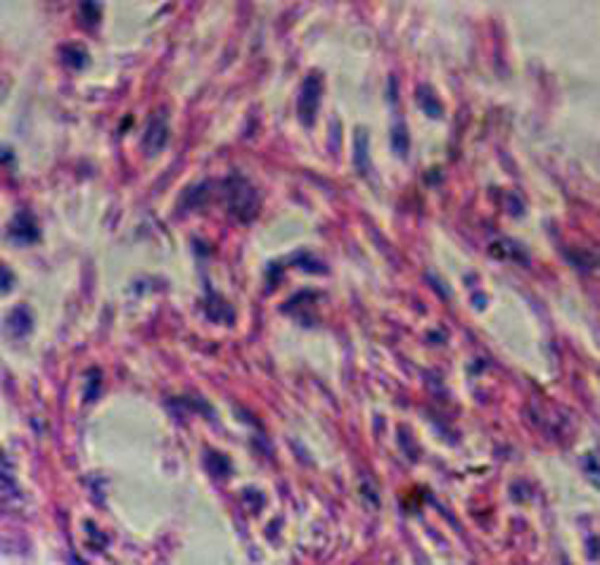
**Tumor section with HE stain (HE × 400)**. Tumor cells are small and round with few processes, darkly-stained round nuclei, rare mitotic figures, and fine chromatin in scattered distribution patterns. Cytoplasm was sparse and perinuclear halos appear empty.

The patient is a 52-year-old man who presented to our division after six months of deteriorating vision associated with metamorphopsia. On physical examination, visual acuity of the left eye was found to be 20/20, whereas vision in the right eye was limited to detection of horizontal motion of the examiner's hand. The anterior and posterior segments of the left eye were normal. The movement of the right eye was smooth, and the anterior segment was normal, but an irregularly shaped solid mass was found in the anterior temporal vitreous cavity, coupled with a localized concave surface in the adjacent retina. B-mode ultrasound examination revealed a mass-like echo of medium intensity roughly distributed within the echo representing the right anterior vitreous cavity. The posterior portion of the vitreous produced a "v"-shaped echo with its cutting-edge connecting to that of the optic disk (Figure 1). Color Doppler ultrasonic examination showed a mass-like echo having a clear boundary and a smooth surface of approximately 1.7 cm × 1.0 cm on the temporal side of the right eye. The structure forming the echo extended to the ciliary process and included a small, irregular anechoic area. A Computed tomography scan revealed an approximately semi-circular, non-homogeneous, high-density area in the right eye extending from the vitreous cavity to the concave surface of the outer ring: The scope of infringement consisted of the area between the retina and the ciliary body. A visible crescent-shaped shadow having a clear border was found in the posterior vitreous (Figure 2). Magnetic resonance imaging demonstrated a block having irregular and unclear edges with a long T1 and short T2 signal area in the lateral ciliary body. A crescent-shaped signal with shorter T1 and short T2 was found in the retinal area. The optic nerve signal in the right eye was normal (Figure 3, 4). The patient was given a preliminary diagnosis of oligodendroglioma of the ciliary body with secondary retinal detachment of the right eye. Using local anesthesia, enucleation was followed by implantation of an artificial right eye. The integrity of the excised eye was preserved; the optic nerve was intact. The temporal side of the sclera was thinning locally and had turned black. Specimens were sent for histopathological examination. HE staining revealed single, small, round tumor cells having few processes, darkly-stained round nuclei, rare mitotic figures, and finely-stained chromatin in scattered distribution patterns. Cytoplasm was sparse and perinuclear halos appeared empty (Figure 5). Immunohistochemistry results were as follows: OLIGO-2 (+), GFAP (-), NF (-), CD57 (focal +), CGA (-), CK (-), S-100 (scattered +), NSE (+/-), D34 (-), HMB45 (-), KI67 (+, <2%). The pathological diagnosis was oligodendroglioma of the ciliary body in the right eye (WHO grade II).

## Disscussion

**Figure 6:**
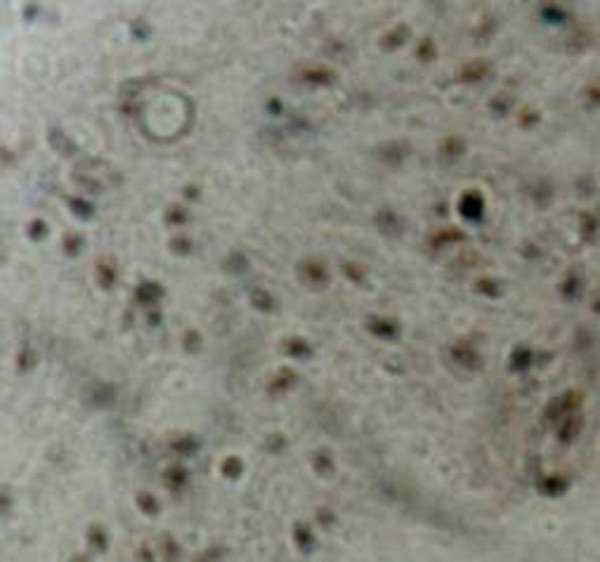
**Immunohistochemical staining of the nucleus was positive for OLIGO-2**. (HE × 200)

**Figure 7:**
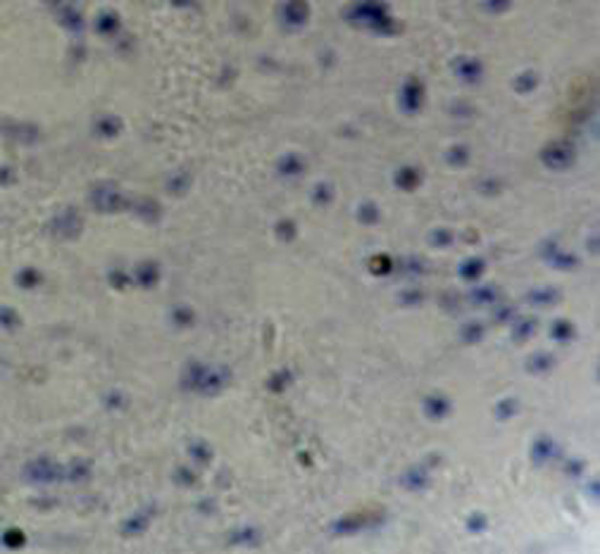
**Immunohistochemical staining of the cytoplasm was negative for GFAP**. (HE × 200)

Oligodendrogliomas, which originate in oligodendrocytes, are relatively rare neuroepithelial tumors, accounting for about 3% of all intracranial tumors and 20% of gliomas [7]. They are more commonly found in adults, and occur mainly in the superficial gray matter of the cerebral hemispheres. For this tumor, 69% of the lesion is located in the frontal lobes and up to 20% of the tumor located in the temporal lobes. Involvement of the hypothalamus and optic chiasm are rarely seen and the tumor is most rare within the eye itself. Oligodendrogliomas with expansive growth of glioma-based and slow growth are relatively benign gliomas (WHO grades I-II). Early diagnosis of ciliary body tumors is difficult because they are well-hidden within the iris. Our patient presented with symptoms secondary to retinal detachment: decreased vision and metamorphopsia. Clinical examination and imaging studies revealed a ciliary body tumor, but definitive diagnosis required pathological examination. The nucleus was positive for OLIGO-2 and the cytoplasm was positive for GFAP on immunohistochemical staining (Figure6, 7). OLIGO-2 expression is relatively specific for oligodendrogliomas [8], and the combination of OLIGO-2 and GFAP expression provides further support for the diagnosis. Since treatment principles for oligodendrogliomas of the ciliary body should be consistent with those for intracranial gliomas, complete resection could be expected to improve long-term survival. In our patient the volume of the oligodendroglioma had resulted in significant enlargement of the ciliary body. We hypothesize that patient survival might be higher in these patients following enucleation.

The pathogenesis of oligodendrogliomas of the ciliary body should be consistent with that for intracranial gliomas, which originate from neural stem cells. Neural stem cells are a group of cells that differentiate into neurons, glial cells and oligodendrocytes [9]. They are found in the brain tissue of human embryos, and they persist into adulthood. Retinas differentiate from the neuroectoderm and as such can be considered extensions of the brain. Neural stem cells may be found in the retina [10], and in 2004 Coles found that retinal stem cells are located in the lateral half of the iris, in the pars plana and in ciliary processes [11]. As a specific subset of neural stem cells, retinal stem cells are pluripotent. Like mitogen-responsive neural stem cells, retinal stem cells can be induced to differentiate into neurons and glial cells. The eyes of adult Ahmad rats, when placed into a mitogenic medium (containing bFGF and EGF), formed a single-cell culture, thereby confirming that neural precursor cells of ciliary pigmented epithelium can differentiate into star-shaped glial cells and oligodendrocytes [12].

Our case provides further confirmation that gliomas might occur wherever neural stem cells exist. Malignant gliomas derive from transformed neural stem cells. Oligodendrogliomas and astrocytomas of the ciliary body originate from oligodendrocytes and astrocytes that have differentiated from retinal stem cells. Ciliary retinal stem cells have the same properties of mobility and pluripotentiality as neural stem cells. When stimulated by carcinogenic factors, retinal stem cells can give rise to tumors of a variety of cell types. It is possible for stem cells derived from retinal ciliary epithelium to be well-differentiated and mature enough to replace dead retinal neurons when surgically transplanted[13-15]. Furthermore, ciliary retinal stem cells, when placed into an environment of retinal growth and development, have more cell differentiation potential for retinal transplantation than do other types of stem or progenitor cells. Therefore, finding an oligodendroglioma of the ciliary body provides evidence for the presence of retinal stem cells in this anatomical location. This knowledge may ultimately lead to novel approaches to treatment of numerous degenerative diseases such as retinitis pigmentosa, the advanced stages of glaucoma, age-related macular degeneration, and optic nerve atrophy.

## Conclusion

Definitive diagnosis of oligodendroglioma of the ciliary body can be made only after results of pathological and immunohistochemical analyses are known. Although extremely rare, preoperatively oligodendroglioma should be included in the differential diagnosis of ciliary body tumors.

## Competing interests

The authors declare that they have no competing interests.

## Authors' contributions

QG, SBS and QY are the main authors. SPX, GDC and QLG have been involved in drafting the manuscript and revising it for intellectual content.JH provided figures.All Authors have given approval to the final version of the work.

## Pre-publication history

The pre-publication history for this paper can be accessed here:

http://www.biomedcentral.com/1471-2407/10/579/prepub
